# Recommendations for the adjuvant use of the poly-antibiotic–resistant probiotic *Bacillus clausii* (O/C, SIN, N/R, T) in acute, chronic, and antibiotic-associated diarrhea in children: consensus from Asian experts

**DOI:** 10.1186/s40794-020-00120-4

**Published:** 2020-10-23

**Authors:** Jo-Anne De Castro, Dhanasekhar Kesavelu, Keya Rani Lahiri, Nataruks Chaijitraruch, Voranush Chongsrisawat, Pramod Prabhakar Jog, Yun Haw Liaw, Gia Khanh Nguyen, Thi Viet Ha Nguyen, Uday Ananth Pai, Huu Nguyet Diem Phan, Seng Hock Quak, Pornthep Tanpowpong, Mary Jean Guno

**Affiliations:** 1 De La Salle University Medical Center, Dasmariñas, Cavite Philippines; 2 Apollo Children Hospital, Chennai, India; 3 D. Y. Patil Medical College & Hospital, Navi Mumbai, India; 4 Faculty of Medicine, Chulalongkorn University and King Chulalongkorn Memorial Hospital, Bangkok, Thailand; 5 D. Y. Patil Medical College, Pune, India; 6 KPJ Sabah Specialist Hospital, Kota Kinabalu, Sabah Malaysia; 7 Vietnam Pediatric Association, Hanoi, Vietnam; 8 Hanoi Medical University, Hanoi, Vietnam; 9 Pai’s Clinic, Chembur, Mumbai, India; 10 University of Medicine and Pharmacy of Ho Chi Minh City, Ho Chi Minh City, Vietnam; 11 National University Hospital, Singapore, Singapore; 12 Faculty of Medicine Ramathibodi Hospital, Mahidol University, Bangkok, Thailand; 13 Institute of Pediatrics, Section of Pediatric Gastroenterology, Hepatology and Nutrition, The Medical City, Pasig City, Philippines

**Keywords:** Acute viral diarrhea, Antibiotic-associated diarrhea, *Bacillus clausii*, *Clostridium difficile*-associated diarrhea, Chronic diarrhea, Gastroenteritis, *Helicobacter pylori* infection, Pediatric diarrhea, Probiotics, Spores

## Abstract

This paper proposes recommendations for probiotics in pediatric gastrointestinal diseases in the Asia-Pacific region. Evidence-based recommendations and randomized controlled trials in the region are included. Cultural aspects, health management issues and economic factors were also considered. Final recommendations were approved by utilizing a modified Delphi process and applying the Likert scale in an electronic voting process. *Bacillus clausii* was recommended as an adjunct treatment with oral rehydration solution for acute viral diarrhea. *B. clausii* may also be considered for prevention of antibiotic-associated diarrhea, *Clostridium difficile*-induced diarrhea, and as adjunct treatment of *Helicobacter pylori.* There is insufficient evidence for recommendations in other conditions. Despite a diversity of epidemiological, socioeconomical and health system conditions, similar recommendations currently apply to most Asia-Pacific countries. Ideally, these need to be validated with local randomized-controlled trials.

## Introduction

Diarrhea, although a preventable disease, remains a major cause of morbidity and mortality in children worldwide, resulting in 525,000 deaths per year among those younger than 5 years [1]. Globally, the most important enteric pathogen is rotavirus [2]. The World Health Organization (WHO) recommends treatment of acute childhood diarrhea with oral rehydration salts (ORS) and continued feeding for the prevention and treatment of dehydration, as well as zinc supplementation to shorten the duration and severity of the diarrheal episode. Prospective clinical trials conducted found *Bacillus clausii* (O/C, SIN, N/R, T) to be effective and safe in the treatment and prevention of acute diarrhea [1].

*B. clausii* (O/C, SIN, N/R, T) are rod-shaped, nonpathologic spore-forming bacteria that is able to survive gastric transit, resist high temperature, acid and bile salt, and colonize the intestine even in the presence of antibiotics. The designations of these bacterial strains are derived from the spores’ resistance to diverse antibiotics: O/C, resistant to chloramphenicol; N/R, resistant to novobiocin and rifampin; SIN, resistant to neomycin and streptomycin; T, resistant to tetracycline [1]. *B. clausii* with four strains, O/C, SIN, N/R and T, is marketed as suspension of spores in vials, capsule and powder sachet for oral usage, and is indicated for the treatment and prevention of intestinal dysmicrobism and subsequent endogenous avitaminosis, and acute and chronic gastrointestinal disorders in infants [3]. Poly-antibiotic resistance is attributed to the bacteria’s multiple strains, with each strain having its own set of resistance to a wide range of antibiotics and this allows it to be used as adjunct with other antibiotics [1]. Moreover, *B. clausii* (O/C, SIN, N/R, T) demonstrates a low level of intraspecific genome activity and exhibits a high degree of genomic conservation for the last 25 years [4].

### Need for probiotic recommendations in the Asian region

The interaction between diet and gut microbiota, and ultimately their effect on human health, has been the subject of huge interest and research. However, this relationship still needs to be fully characterized, particularly in case of the Asian population. While much of the function of the gut microbiome remains to be fully elucidated, it is established that the microbiota plays an important role in maintaining health [5]. The composition of microbiota is altered in certain disease states, including enteric infections, *Helicobacter pylori* and *Clostridium difficile* infection, and antibiotic-associated diarrhea (AAD), resulting in a state of dysbiosis. Dysbiosis is related to various important pathologies and many therapeutic strategies aimed at restoring the balance of the intestinal ecosystem have been implemented. These strategies include the administration of probiotics and prebiotics.

Mortality and morbidity rates remain high despite global efforts to treat diarrhea. This necessitates continued efforts on improving diarrhea management and diarrhea prevention.

This consensus paper captures an up-to-date overview, from a community of invited key opinion leaders across Asia, based on pure evidence from published literature around the scope of recommendation.

### Target audience and contents

The intended target audience of this document includes pediatricians, primary care physicians and all healthcare professionals involved in the management of pediatric diarrhea. The document includes 11 recommendations across the following sections: Probiotics as adjunct treatment in diarrhea, *B. clausii* (O/C, SIN, N/R, T) in acute diarrhea, *B. clausii* (O/C, SIN, N/R, T) in chronic diarrhea, and *B. clausii* (O/C, SIN, N/R, T) in AAD.

### Objectives

The primary objective is to develop a consensus document that would serve as a reference for the management of pediatric diarrhea in the Asian region, focusing on the use of the poly-antibiotic–resistant *Bacillus clausii* (O/C, SIN, N/R, T) in light of new evidence of its efficacy and safety as an adjuvant in the treatment of acute diarrhea, chronic diarrhea, and AAD.

## Materials and methods

A modified Delphi process [6], a very common process that involves the combined insights of an expert panel, was utilized in developing the consensus recommendations. This was done by conducting two expert panel meetings: 1) First meeting on 28 June 2019 in Manila, Philippines and 2) Second meeting on 27 September 2019 in Ho Chi Minh City, Vietnam.

### Assembly of the Asian expert panel

An expert panel was formed by inviting 14 medical experts from various countries, including Philippines, India, Vietnam, Singapore, Thailand and Malaysia. The members of the expert panel included pediatricians, pediatric gastroenterologists and a pediatric infectious disease specialist who are involved in the management of pediatric diarrhea. Initially, 11 experts attended the first panel meeting in Manila, Philippines and three additional experts joined the second panel meeting in Ho Chi Minh City, Vietnam. All members of the expert panel contributed to the discussions and were actively involved in every phase of the consensus recommendation process. No representative from pharmaceutical companies was involved in any part of the recommendation development process and all the experts were independent in making their choice.

The first meeting was convened to discuss contemporary approaches to, and review the latest evidence in, the management of pediatric diarrhea, and present real-world experiences and consolidate best practices on the use of *B. clausii* in the management of pediatric diarrhea. The second meeting was convened to present and discuss the evidence that supports the consensus statements on the use of *B. clausii* in the management of acute diarrhea, chronic diarrhea, and AAD; to discuss, agree and finalize the consensus recommendations on the use of *B. clausii* in the management of acute diarrhea, chronic diarrhea, and AAD; and to finalize the next steps in the development of the consensus paper.

### Scope of recommendations

The members of the expert panel developed recommendations pertaining to the use of probiotics as adjunct treatment in diarrhea, and the use of *B. clausii* in acute diarrhea, chronic diarrhea, and AAD.

### Search strategy

The expert panel was divided into four groups based on the following topics: 1) Probiotics as adjunct treatment in diarrhea, 2) *Bacillus clausii* (O/C, SIN, N/R, T) in acute diarrhea, 3) *Bacillus clausii* (O/C, SIN, N/R, T) in chronic diarrhea, and 4) *Bacillus clausii* (O/C, SIN, N/R, T) in AAD. Each group was asked to search and review relevant literature based on the initial consensus statements developed, and to augment and consolidate the evidence. The approach was to review MEDLINE, Embase and Google Scholar, and to review the initially drafted consensus statements which contain some suggested references.

### Search criteria

The scope of the literature search of the Probiotics group was limited to literature published within a 10-year time frame (2009 to 2019), while the other groups were asked to include literature published within a wider time frame (from the 1980s to present) to gather more information given that *B. clausii* has been available in the market for quite a long time.

### Evidence synthesis

Documents were prepared based on the initial consensus statements drafted from the first expert panel meeting. This was followed by a comprehensive literature search and review and collation of supporting evidence. The members of the expert panel did not grade the evidence; the grade and strength of evidence presented were based on the references cited.

### Development of consensus recommendations

Members of the first expert panel meeting summarized the evidence and consensus statements. These were presented at the second expert panel meeting. After each discussion, each member of the expert panel voted on the proposed consensus statements. A 5-point Likert scale [7] (strongly agree, agree, neither agree or disagree, disagree, or strongly disagree) was utilized via electronic voting. All members were involved in scrutinizing the content and form of each statement, as well as how the statement was worded. Disagreements were resolved through discussion of suggested modifications, rewording and rephrasing of consensus statements, and another round of voting. The members agreed that a consensus is achieved when, combining the votes for strongly agree and agree, a vote of at least 80% is reached. The results of the consensus voting are summarized in Appendix, Table 8. The accepted statements were finalized as the expert panel’s consensus recommendations.

## Results

Each recommendation is presented together with relevant evidence and accompanied by supporting text, which is structured as follows.

- *Summary of evidence linked to recommendation statement*
- Summary of the evidence presented
- Table/s to present the data from the evidence
- Special comment or expert opinion relevant to the recommendation presented

## Recommendations

A summary of the recommendations is presented in Table 1. It should be noted that the recommendations are categorized into four sections: 1) Probiotics as adjunct treatment in diarrhea, 2) *Bacillus clausii* (O/C, SIN, N/R, T) in acute diarrhea, 3) *Bacillus clausii* (O/C, SIN, N/R, T) in chronic diarrhea, and 4) *Bacillus clausii* (O/C, SIN, N/R, T) in AAD. These sections may, thus, be referenced separately and individually.

**Table 1:** Asian experts’ recommendations on the use of probiotics and *Bacillus clausii* (O/C, SIN, N/R, T) in the management of pediatric diarrhea

**Section 1: Probiotics as adjunct treatment in diarrhea**	
1. Acute viral diarrhea is the best-established indication for probiotics administration in childhood. Probiotics have a promising role in the treatment of acute viral diarrhea.	
2. Evidence has accumulated on the efficacy of probiotics in reducing the duration and severity of acute diarrhea in children.	
3. Probiotics administration may be considered as an adjunct therapy for the prevention of antibiotic-associated diarrhea.	
4. Probiotics significantly reduce the risk of *Clostridium difficile*-associated diarrhea in adults and children.	
**Section 2:** ***Bacillus clausii*** **(O/C, SIN, N/R, T) in acute diarrhea**	
1. *Bacillus clausii* (O/C, N/R, SIN, TETRA) may be considered as adjunct to ORS and zinc in acute childhood diarrhea.	
2. *Bacillus clausii* has been found to be safe in clinical trials conducted in Asian children with acute diarrhea.	
3. Strain-specific poly-antibiotic–resistant *Bacillus clausii* is efficacious in reducing the duration and frequency of diarrhea, hospital stay, and financial burden.	
**Section 3:** ***Bacillus clausii*** **(O/C, SIN, N/R, T) in chronic diarrhea** 1. There is very limited evidence supporting the use of probiotics for the management of chronic/persistent diarrhea in children.	
**Section 4:** ***Bacillus clausii*** **(O/C, SIN, N/R, T) in antibiotic-associated diarrhea**	
1. Probiotics administration may be considered for the prevention of antibiotic-associated diarrhea (AAD).	
2. Physicians should evaluate the risk factors for the occurrence of AAD or *Clostridium difficile*-associated diarrhea, such as the class of antibiotics, duration of antibiotic treatment, need for hospitalization, age, comorbidities, and previous episodes of AAD or *C. difficile*-associated diarrhea when considering probiotics for prevention of AAD in children.	
3. *Bacillus clausii* can be used as co-adjuvant therapy for *Helicobacter pylori* eradication.	

### Section 1: probiotics as adjunct treatment in diarrhea

#### Recommendation 1. Acute viral diarrhea is the best-established indication for probiotics administration in childhood. Probiotics have a promising role in the treatment of acute viral diarrhea

##### Summary of evidence linked to recommendation statement

The 2014 European Society for Pediatric Gastroenterology, Hepatology, and Nutrition/European Society for Pediatric Infectious Diseases (ESPGHAN/ESPID) evidence-based guidelines for the management of acute gastroenteritis in children in Europe [8] provided strong recommendations for *Lactobacillus rhamnosus* GG (LGG) and *Saccharomyces boulardii* in the treatment of acute gastroenteritis (AGE) based on reviewed meta-analyses (Table 2). The 2014 Use of probiotics for management of acute gastroenteritis: A position paper by the ESPGHAN Working Group for Probiotics and Prebiotics [9] likewise recommended that LGG, *S. boulardii* and *Lactobacillus reuteri* may be considered as an adjunct in the management of children with AGE.

**Table 2:** Probiotics with a positive recommendation for treating acute gastroenteritis

Strain(s)	Quality of evidence	Recommendation	Dose
*Lactobacillus rhamnosus* GG (LGG)	Low	Strong	≥10^10^ CFU/day (typically 5–7 days)
*Saccharomyces boulardii*	Low	Strong	250–750 mg/day (typically 5–7 days)
*Lactobacillus reuteri* DSM 17938	Very low	Weak	10^8^ -4 × 10^8^/day (typically 5–7 days)
Heat-killed *Lactobacillus acidophilus* LB	Very low	Weak	Minimum 5 doses of 10^10^ CFU for 48 h, maximum 9 doses of 10^10^ CFU for 4.5 days

*CFU* Colony-forming units

The Asia-Pacific regional guidelines [2] likewise proposed that LGG, *L. reuteri* and *S. boulardii* should be strongly recommended for acute gastroenteritis (Table 3).

**Table 3:** Proposed probiotic recommendations for the Asia-Pacific region

Disease	Recommendation	Strain	Grade	Strength
Acute gastroenteritis	Should be recommended	*S. boulardii* *L. rhamnosus GG* *L. reuterii*	Moderate quality	Strong
AAD	May be considered	*L. rhamnosus GG* *S. boulardii*	Moderate quality	Strong
CDAD	May be considered	*S. boulardii*	Low quality	Weak

*AAD* Antibiotic-associated diarrhea, *CDAD Clostridium difficile*-associated diarrhea

Based on the large number of high-quality randomized, controlled trials (RCTs), LGG and *S. boulardii* were strongly recommended as an adjunct to oral rehydration therapy in children of the Asia-Pacific region [2], based on all the summarized recommendations of professional societies (ESPGHAN/ESPID in Europe, American Academy of Pediatrics in the United States, Latin American group and the World Gastroenterology Organisation [WGO]) (Table 4).

**Table 4:** Recommendations for use of probiotics in childhood diseases by geographic region

Diseases	Use	Europe	United States	Latin America	WGO
Acute gastroenteritis	T	*L. rhamnosus GG,* *S. boulardii,* *L. reuteri*	*L. rhamnosus GG,* *S. boulardii*	*L. rhamnosus GG,* *S. boulardii,* *L. reuteri*	*S. boulardii,* *L. rhamnosus GG*, Indian Dahi
AAD	P	*L. rhamnosus GG,* *S. boulardii*	*L. rhamnosus GG,* *S. boulardii*	*L. rhamnosus GG,* *S. boulardii*	S*. boulardii,* *L. rhamnosus GG*, *B. lactis* Bb12+ *S. thermophilus, L. rhamnosus* strains E/N, Oxy and Pen
CDAD	P	*S. boulardii*			

*AAD* Antibiotic-associated diarrhea, *CDAD Clostridium difficile*-associated diarrhea, *P* Prevention, *T* Treatment, *WGO* World Gastroenterology Organisation

Adapted from: Cameron D, et al. World J Gastroenterol. 2017;23(45):7952–7964

With probiotics being strain-specific, it should be emphasized that conclusions regarding a particular strain cannot be extrapolated to another strain. The quality of evidence is taken into account only by the number of RCTs.

#### Recommendation 2. Evidence has accumulated on the efficacy of probiotics in reducing the duration and severity of acute diarrhea in children

##### Summary of evidence linked to recommendation statement

The ESPGHAN/ESPID guideline [8] states that administration of effective probiotic strains reduces the duration of hospital stay and may be considered in children admitted for AGE (II, B) (strong recommendation, low-quality evidence). Administration of probiotics in hospitalized children reduced the mean length of hospitalization by 1.12 days (95% confidence interval [CI], − 1.16 to 0.38). Compelling evidence in support of effective strains is available for LGG and *S. boulardii.* In this ESPGHAN/ESPID paper, *B. clausii* (O/C, SIN, N/R, T) was included as “low quality of evidence” due to the lack of RCTs.

The duration, frequency and consistency are the three components measured in diarrhea. There were some studies that showed probiotics reduced the duration of diarrhea. A systematic review and meta-analysis of six RCTs (Canani, et al. 2007; Maugo. 2012; Urtula, Dacula. 2008; Lahiri. 2008; Lahiri, Jadhav, et al. 2015; and Lahiri, D’Souza, et al. 2015) involving 898 patients showed a significant reduction in the duration of acute diarrhea in children in the *B. clausii* group compared with the ORS with or without zinc supplementation group (mean difference = − 9.12 h; 95% CI, − 16.49 to − 1.75; *p* = 0.015) [1].The results of the six studies are summarized in Appendix, Table 9).

#### Recommendation 3. Probiotics administration may be considered as an adjunct therapy for the prevention of antibiotic-associated diarrhea

##### Summary of evidence linked to recommendation statement

The prevalence of AAD varies depending on the criteria used to diagnose it; in children, however, it ranges from approximately 5 to 40%. Evidence from several meta-analyses has consistently shown that most of the tested probiotics significantly reduce the risk of AAD in the general (mainly adult) population. A 2012 meta-analysis [10] of pooled data from 63 RCTs involving almost 12,000 participants indicated a statistically significant reduction in the risk of AAD in the probiotic groups compared with the control groups (relative risk [RR], 0.58; 95% CI, 0.50 to 0.68; *p* < 0.001).

Pooled evidence [10] suggests that probiotics are associated with a reduction in AAD. However, more research is needed to determine which probiotics are associated with the greatest efficacy in AAD.

In the 2018 Consensus and contentious statements on the use of probiotics in clinical practice: A South East Asian Gastro-neuro Motility Association (SEAGMA) working team report [11], the expert panel agreed that probiotics decrease the incidence of AAD in children. The evidence comprises a meta-analysis of 22 studies that showed probiotics decreased the incidence of AAD in children versus placebo (8% vs 19%).

The 2004 *Bacillus clausii* therapy to reduce side-effects of anti-*Helicobacter pylori* treatment: Randomized, double-blind, placebo-controlled trial [12] involving 120 *H. pylori*-positive patients who received standard 7 days triple therapy (rabeprazole 20 mg twice a day [BID], clarithromycin 500 mg BID, amoxicillin 1 g BID) and *B. clausii* (2 × 10^9^ spores three times a day [TID]) for 14 days (group A), or standard triple therapy and placebo for 14 days (group B) showed that the incidence of diarrhea related to anti-*H. pylori* antibiotic therapy was significantly lower in group A compared with group B (per protocol [PP], *p* < 0.05; intention-to-treat [ITT], *p* < 0.01) (Table 5).

**Table 5:** Incidence of diarrhea during the treatment period

Symptoms	Per protocol population			Intention-to-treat population		
	***Bacillus clausii*** (%)	Placebo (%)	RR; 95% CI	***Bacillus clausii*** (%)	Placebo (%)	RR; 95% CI
Diarrhea						
- First week	10	30	0.333; 0.13–0.85*	9.3	30.8	0.301; 0.12–0.76**
- Second week	4	8	0.5; 0.10–2.61	3.7	9.6	0.385; 0.08–1.90

*CI* Confidence interval, *RR* Relative risk

* *p* < 0.05; ** *p* < 0.01

Adapted from: Nista EC, et al. Aliment Pharmacol Ther. 2004;20:1181–1188

#### Recommendation 4. Probiotics significantly reduce the risk of *Clostridium difficile*-associated diarrhea in adults and children

##### Summary of evidence linked to recommendation statement

The group generally agreed that probiotics significantly reduce the risk of *Clostridium difficile*-associated diarrhea (CDAD) in adults and children. The 2017 Probiotics for the prevention of *Clostridium difficile*-associated diarrhea in adults and children [13] systematic review and meta-analysis of 31 RCTs involving 8672 patients suggests that probiotics are effective for preventing CDAD (number needed to treat for an additional beneficial outcome = 42 patients; 95% CI, 32 to 58). The ESPGHAN working group [8] proposed that probiotics (LGG and *S. boulardii*) may be considered for the prevention of CDAD based on the evaluation of individual cases (see Table 2).

The 2004 *Bacillus clausii* probiotic strains: Antimicrobial and immunomodulatory activities [14] showed that *B. clausii* strains secrete antimicrobial substances and this was observed during stationary growth which coincided with sporulation. These substances were active against gram-positive bacteria which includes *Staphylococcus aureus*, *Enterococcus faecium* and *C. difficile*. The 2016 Secreted compounds of the probiotic *Bacillus clausii* strain O/C inhibit the cytotoxic effects induced by *Clostridium difficile* and *Bacillus cereus* toxins [15], studied the ability of the compounds secreted by *B. clausii* strain O/C – a bacteriocin and an alkaline serine M-protease called *clausin* – against the cytotoxic effects induced by *C. difficile* and *B. cereus* through co-incubation of toxic culture supernatants. Results showed that the *B. clausii* supernatant completely prevented the toxin-induced damage in Vero and Caco-2 cells and prevented the hemolytic effects of *B. cereus*.

The 2015 systematic review with meta-analysis: *Saccharomyces boulardii* in the prevention of antibiotic-associated diarrhoea [16] of 21 RCTs, of which 16 are new trials, involving 4780 participants showed that *S. boulardii* reduced the risk of AAD from 18.7 to 8.5% in patients treated with antibiotics compared with placebo (RR, 0.47; 95% CI, 0.38 to 0.57; number needed to treat [NNT] = 10; 95% CI, 9 to 13). In children, *S. boulardii* reduced the risk from 20.9 to 8.8% (6 RCTs, *n* = 1653; RR, 0.43; 95% CI, 0.3 to 0.6; NNT = 9; 95% CI, 7 to 12). Moreover, in the subgroup analysis, *S. boulardii* reduced the risk of CDAD in children (2 RCTs, *n* = 579; RR, 0.25; 95% CI, 0.08 to 0.73) but not in adults (9 RCTs, *n* = 1441; RR, 0.80; 95% CI, 0.47 to 1.34).

The 2017 Timely use of probiotics in hospitalized adults prevents *C. difficile* infection (CDI): A systematic review and meta-regression analysis [17] of 19 studies involving 6261 subjects showed that probiotics were significantly more effective if given closer to the first antibiotic dose, with a decrement in efficacy for every day of delay in starting probiotics (*p* = 0.04). Probiotics given within 2 days of antibiotic initiation produced a greater risk reduction for *C*DI (RR, 0.32; 95% CI, 0.22 to 0.48; I^2^ = 0%) compared with later administration (RR, 0.70; 95% CI, 0.40 to 1.23; I^2^ = 0%) (*p* = 0.02). There was no increased risk for adverse events among patients given probiotics.

Moreover, the *B. clausii* strain O/C produces an antimicrobial substance, called *clausin*, that is active against the gram-positive bacteria *C. difficile* [15].

### Section 2: *Bacillus clausii* (O/C, SIN, N/R, T) in acute diarrhea

#### Recommendation 5. *Bacillus clausii* (O/C, N/R, SIN, TETRA) could be considered as adjunct to ORS and zinc in acute childhood diarrhea

##### Summary of evidence linked to recommendation statement

The 2019 *Bacillus clausii* as adjunctive treatment for acute community-acquired diarrhea among Filipino children: A large-scale, multicenter, open-label study (CODDLE) [18] evaluated 2916 children (median age, 2 years) with community-acquired diarrhea of < 48 h (either AAD or viral diarrhea) treated with *B. clausii* (O/C, SIN, N/R, T) 1 to 2 vials (2 billion spores of *B. clausii* O/C, SIN, N/R, T strains/5 mL vial) per day for 5 to 7 days. Concomitant treatment, such as ORS, zinc supplements and antibiotics prescribed for other conditions, was permitted and recorded; other probiotics and antidiarrheal drugs were prohibited. The study showed that, with *B. clausii* treatment, the mean duration of diarrhea was 3 days in 52.7% of patients. In the AAD subgroup, the mean duration of diarrhea was shorter in children treated with zinc compared with children without zinc supplementation (3.2 ± 1.4 d vs 3.3 ± 1.3 d). In contrast, in the viral diarrhea subgroup, the mean duration of diarrhea was longer in zinc-treated children compared with children without zinc supplementation (3.6 ± 1.4 d vs 3.3 ± 1.3 d). Moreover, the mean diarrheal episodes significantly decreased from a median of 5 episodes/day at baseline to 1 episode/day starting from the fifth day of treatment (*p* < 0.001). *B. clausii* (O/C, SIN, N/R, T) also significantly reduced the mean number of stools per day from 5.2 ± 2.0 stools at baseline to 1.3 ± 0.6 stools after 7 days of treatment (*p* < 0.001).

The 2017 Role of *Saccharomyces boulardii* and *Bacillus clausii* in children with acute diarrhea: A randomized controlled trial [19] involving hospitalized infants and children (6 months to 3 years of age) grouped patients to receive either ORS and zinc per WHO standard protocol for acute diarrhea (group A); ORS, zinc and *S. boulardii* (250 mg BID × 5 days) (group B); or ORS, zinc and *B. clausii* (2 × 10^9^ CFU/mL BID × 5 days) (group B) and compared the reduction in the duration of diarrhea. The study showed a significant reduction in the mean duration of diarrhea between groups A and B (107.25 ± 24.37 h vs 84.71 ± 21.49 h; *p* < 0.05), and between groups A and C (107.25 ± 24.37 h vs 89.46 ± 24.20 h; *p* < 0.05), which means that *S. boulardii* and *B. clausii* could be used as adjunct to ORS and zinc in the treatment of acute childhood diarrhea.

#### Recommendation 6. *Bacillus clausii* Has been found to be safe in clinical trials conducted in Asian children with acute diarrhea

##### Summary of evidence linked to recommendation statement

In the 2019 CODDLE study [18], the mean duration of treatment with *B. clausii* (O/C, SIN, N/R, T) was 5.8 ± 1.3 days with a median total dose of 10 vials (2 vials/day). The treatment was well tolerated with only three reported adverse events, which includes vomiting, erythematous rashes and change in stool color, all of which were mild-to-moderate in severity. The study documented the good safety and tolerability profile of *B. clausii* (O/C, SIN, N/R, T) in the management of acute childhood community-acquired diarrhea.

In the meta-analysis by Ianiro, et al. [1], the studies did not report any serious adverse effects related to *B. clausiii* (O/C, SIN, N/R, T).

#### Recommendation 7. Strain-specific poly-antibiotic–resistant *Bacillus clausii* is efficacious in reducing the duration and frequency of diarrhea, hospital stay, and financial burden

##### Summary of evidence linked to recommendation statement

Revisiting studies [1] on probiotics showed the safety and efficacy of probiotics as adjunct to ORS and zinc (Table 6).

**Table 6:** Safety and efficacy of *Bacillus clausii* as adjunct to ORS and zinc

Studies	Study design	Number of patients/Age	Outcomes	Results
Canani et al. (2007, Italy)	Prospective, multicenter, single-blind, RCT	100/92 Median 18 months	Duration, frequency and consistency of stool, hospital stay, safety and tolerability	All other outcomes were also similar in both groups. *Bacillus clausii* was well tolerated, with no observed AEs
Lahiri. (2008, India)	Phase III, controlled, open-label, multicenter, parallel-group, comparative, RCT	132/132 Mean 1.6 years	Duration, frequency and consistency of stool, vomiting, tolerability and AE	Duration and frequency of stool lower in experimental group
Lahiri, et al. (2015, India)	Open-label, prospective, RCT	69/62 6 months to 12 years	Duration and frequency of diarrhea, hospitalization and cost	Duration: 22.64 h vs 47.05 h; Hospital stay: 2.78 d vs 4.30 d (*p* < 0.01); Cost reduced by 472 Indian rupees
Lahiri, et al. (2015, India)	Open-label, Prospective, RCT	80/80 Up to 6 years	Duration and frequency of diarrhea	Duration: In study group, 22.26 h vs 34.16 h; Frequency of stools, 1.15 vs 1.70 (*p* < 0.05)
Maugo. (2012, Kenya)	Randomized, double blind, placebo-controlled	51/51 6 to 59 months	Duration, frequency of diarrhea and hospitalization	Duration: Shorter in study group, 9.15 h; Frequency of stools: Significant decrease in frequency in study group, on day 3 (2.74 [1.81] motions) vs 3.80 [2.70] motions in placebo group, mean absolute difference = 1.05 motions; *p* = 0.033) and on day 4 (1.45 [1.13] motions vs 2.35 [2.19] motions in placebo group, mean absolute difference = 0.9 motions; *p* = 0.018) vs placebo group
Urtula, Dacula. (2008, Philippines)	Monocentric, RCT	35/35 NR	Duration, frequency of diarrhea and hospitalization	Significant difference in duration (13.92 h); Shorter hospital stay in *B. clausii* group

*AE* Adverse event, *RCT* Randomized, controlled trial, *NR* Not reported, *ORS* Oral rehydration salts

Adapted from: Ianiro G, et al. Nutrients. 2018;10:1074

The WHO recommends treatment of acute childhood diarrhea with ORS and continued feeding for the prevention and treatment of dehydration, as well as zinc supplementation to shorten the duration and severity of diarrhea. It has been suggested that probiotics modulate the immune response and are used as an adjunctive treatment. In terms of efficacy, the response to the dose of 2 billion or 4 billion spores is similar. *B. clausii* contributes to the restoration of intestinal microbial flora, aids in correcting avitaminosis due to antibiotics, and produces antigenic and antitoxic effects which is closely connected with its metabolic action [3]. Moreover, *B. clausii* (O/C, SIN, N/R, T) was found alive in the feces for up to 12 days following a single dose administration of three vials containing 2 × 10^9^ CFU of *B. clausii* spores [20].

To date, literature surveys have yielded positive results of *B. clausii* in reducing the duration and frequency of diarrhea, stool consistency, hospital stay and cost. Well-designed studies have shown reduction in the duration and frequency of diarrhea, and improved consistency of stool. *B. clausii* as adjunct treatment reduced hospital stay and downscaled economic burden.

### Section 3: *Bacillus clausii* (O/C, SIN, N/R, T) in chronic diarrhea

#### Recommendation 8. There is very limited evidence supporting the use of probiotics for the management of chronic/persistent diarrhea in children

##### Summary of evidence linked to recommendation statement

There is very limited evidence supporting the use of probiotics for the management of chronic/persistent diarrhea in children. Most of the experience shared by people have been anecdotal and there is no evidence that probiotics have any adverse effects when used in the long-term for chronic diarrhea. While *B. clausii* (O/C, SIN, N/R, T) is not included in national guidelines, anecdotal experiences have been largely positive, with significant reductions in the duration and frequency of diarrhea.

According to the WHO, 65 to 85% of patients with gastroenteritis improves in 4 weeks. Beyond 4 weeks, the diarrhea is considered chronic. Acute diarrhea is diarrhea lasting for < 5 days, persistent diarrhea is diarrhea lasting for 5 to 14 days, and chronic diarrhea is diarrhea lasting for > 14 days.

Because probiotics are live microorganisms, they are susceptible to reduction in their cell count during product shelf storage. Responsible manufacturers build in overages so that at the end of the product’s shelf-life, it does not fall below the potency declared on the label. Spore-forming probiotic strains, although not as well studied as others, do have the advantage of superior resistance to environmental stress during shelf-life. Probiotic products on the market have been shown in some cases to fail to meet label claims regarding the numbers and types of variable microbes present on the product [21].

### Section 4: *Bacillus clausii* (O/C, SIN, N/R, T) in antibiotic-associated diarrhea

#### Recommendation 9. Probiotics administration may be considered for the prevention of antibiotic-associated diarrhea (AAD)

##### Summary of evidence linked to recommendation statement

In the prevention of AAD, there is increasing evidence on the efficacy of *B. clausii* in children who are receiving antibiotic therapy. The 1980 Clinical experience with *Bacillus subtilis* in children treated with antibiotics (Puddu study) [22] evaluated 93 Italian children (3 to 14 years of age) and compared the effects of adding *B. subtilis* (now *B. clausii*) (4 vials/day per orem containing 1 billion spores) to antibiotic treatment (group A) versus antibiotic alone (group B) on the incidence of gastrointestinal (GI) problems in children treated with antibiotics for middle ear or throat infection, and nasal and paranasal infections. The study showed that patients in group A had lesser GI problems (11.0% vs 37.5%) (Table 7).

**Table 7:** Incidence of gastrointestinal problems in children treated with antibiotics and *Bacillus subtilis* (group A) versus children treated with antibiotics alone (group B)

	No. of children	Nausea	Vomiting	Abdominal pain	Diarrhea	Aphthae	Total
Group A	45	2	–	2	–	–	5
Group B	48	2	1	9	5	1	18

* χ^2^ = 7.3; *p* < 0.01

The 2004 *Bacillus clausii* therapy to reduce side-effects of anti-*Helicobacter pylori* treatment: Randomized, double-blind, placebo-controlled trial [12] involving 120 *H. pylori*-positive patients who received standard 7 days triple therapy (rabeprazole 20 mg twice a day [BID], clarithromycin 500 mg BID, amoxicillin 1 g BID) and *B. clausii* (2 × 10^9^ spores three times a day [TID]) for 14 days (group A), or standard triple therapy and placebo for 14 days (group B) showed that the incidence of diarrhea related to anti-*H. pylori* antibiotic therapy was significantly lower in group A compared with group B (per protocol [PP], *p* < 0.05; intention-to-treat [ITT], *p* < 0.01) (see Table 5).

Thirty-three (33) studies involving 6352 participants were included in the Cochrane Database of Systematic Reviews of 2019 [23] regarding probiotics for the prevention of pediatric AAD. The probiotics assessed include *Bacillus* spp., *Bifidobacterium* spp., *Clostridium butyricum, Lactobacilli* spp., *Lactococcus* spp., *Leuconostoc cremoris, Saccharomyces* spp., or *Streptococcus* spp. either used alone or in combination. The overall evidence suggests a moderate protective effect of probiotics in preventing AAD (NNT = 9; 95% CI, 7 to 13). Evidence suggests that probiotics may moderately reduce the duration of diarrhea by almost 1 day.

#### Recommendation 10. Physicians should evaluate the risk factors for the occurrence of AAD or *Clostridium difficile*-associated diarrhea, such as the class of antibiotics, duration of antibiotic treatment, need for hospitalization, age, comorbidities, and previous episodes of AAD or *C. difficile*-associated diarrhea when considering probiotics for prevention of AAD in children

##### Summary of evidence linked to recommendation statement

The 2014 Clinical characteristics of symptomatic *Clostridium difficile* infection in children: Conditions as infection risks and whether probiotics is effective [24] study involving 43 children (mean age, 6.7 years) who showed either positive *C. difficile* culture or *C. difficile* toxin test results showed that approximately 40% (*n* = 17) had pre-existing GI disorders (eg, Crohn’s disease, allergic and/or eosinophilic colitis), and 60% (*n* = 26) had no history of GI diseases but with other medical conditions that were risk factors for CDI (eg, extra-intestinal infections like otitis media, tonsillitis); 65% (*n* = 28) had a history of antibiotic treatment for > 3 days. The most frequently prescribed antibiotic was amoxicillin-clavulanate (35.7%, *n* = 10/28 who were previously treated with antibiotics), and the most common symptom at the time of CDI diagnosis was diarrhea (72.1%). Probiotics were prescribed in 28 patients with a history of antibiotic treatment for the prophylaxis of CDI. The most common probiotics used were *L. acidophilus* (53.6%, *n* = 15), *B. subtilis* (now *B. clausii*; 46.4%, *n* = 13) and *Streptococcus faecium* (46.4%, *n* = 13).

Some members of the expert panel would usually recommend giving *B. clausii* in patients prescribed with amoxicillin-clavulanate, especially if the patient has a history of previous AAD with the same antibiotic.

#### Recommendation 11. *Bacillus clausii* can be used as coadjuvant therapy for *Helicobacter pylori* eradication

##### Summary of evidence linked to recommendation statement

In the 2004 *Bacillus clausii* therapy to reduce side-effects of anti-*Helicobacter pylori* treatment: Randomized, double-blind, placebo-controlled trial [12], 120 adult patients, free of GI symptoms in the previous 3 months and affected by gastric *H. pylori* infection as confirmed by a ^13^C-urea breath test were randomly assigned to receive either a triple therapy of clarithromycin 500 mg BID, amoxicillin 1 g BID and rabeprazole 20 mg BID for 7 days plus *B. clausii* (O/C, SIN, N/R, T) one vial TID (containing 2 × 10^9^ spores) for 14 days, during eradication therapy and 1 week thereafter (group A), or triple therapy plus a placebo during eradication therapy and 1 week thereafter (group B). The results showed that *H. pylori* eradication was comparable between groups: ITT analysis (*B. clausii* group, 72.2% vs placebo group, 71.15%); PP population (*B. clausii* group, 78% vs placebo group, 74%). There was a significant difference between both groups in both ITT and PP populations in terms of the incidence of nausea, diarrhea and epigastric pain. In the ITT analysis, the relative risk of occurrence of nausea was reduced to half in patients treated with *B. clausii* compared with placebo after 1 week (RR, 0.5; 95% CI, 0.31 to 0.88) and 2 weeks (RR, 0.45; 95% CI, 0.21 to 0.96) of bacteriotherapy. A greater reduction in the risk of diarrhea was observed in the *B. clausii* group compared with the placebo group after 1 week (RR, 0.30; 95% CI, 0.12 to 0.76) and 2 weeks (RR, 0.38; 95% CI, 0.08 to 1.9). The relative risk of occurrence of epigastric pain after 1 week was 0.68 (95% CI, 0.48 to 0.97); the incidence of vomiting, constipation and skin rash were also lower in the *B. clausii* group compared with the placebo group, although the differences were not statistically significant. The individual patients’ overall assessment of tolerability in the ITT population was better in the *B. clausii* group compared with the placebo group. The difference was statistically significant after 2 weeks of treatment (*p* < 0.05).

The 2017 Probiotics in 14-day triple therapy for Asian pediatric patients with *Helicobacter pylori* infection: A network meta-analysis [25] of 17 RCTs compared the efficacy and safety of probiotics supplemented in 14-day triple therapy in Asian pediatric patients. It was observed that the probiotic-supplemented 14-day triple therapy significantly increased *H. pylori* eradication rates (RR, 1.16; 95% CI, 1.07 to 1.26) and reduced the incidence of total side effects (RR, 0.40; 95% CI, 0.34 to 0.48) compared with placebo. However, further direct evidence is needed to warrant it.

The 2007 Meta-analysis: The effect of supplementation with probiotics on eradication rates and adverse events during *Helicobacter pylori* eradication therapy [26] review suggests that supplementation with probiotics could be effective in increasing eradication rates of anti-*H. pylori* antibiotic regimens and could be considered helpful for patients with eradication failure.

The mechanism by which *B. clausii* is used as coadjuvant therapy for *H. pylori* eradication is not very well understood in the pediatric population. The expert panel agreed that there should be more studies related to *H. pylori*.

## Discussion

Based on the initial consensus statements and evidence presented, 11 recommendations on the use of probiotics and *Bacillus clausii* (O/C, SIN, N/R, T) have been made. The expert panel utilized a modified Delphi process which involved two face-to-face meetings. Through the modified Delphi process, the expert panel was able to discuss the latest evidence on the use of probiotics in the management of pediatric diarrhea and develop consensus recommendations that would serve as a reference for the management of pediatric diarrhea in the Asian region. The Delphi process is a well validated and widely adopted method for systematically assessing and organizing expert opinion. Developed by the RAND corporation® in the 1950s, it is based on the principle that forecasts or decisions from a structured group are more accurate. The method entails a group of experts who anonymously reply to questionnaires and subsequently receive feedback in the form of a statistical representation of the “group response”, after which the process repeats itself. The goal is to reduce the range of responses and arrive at something closer to an expert consensus.

### Expert guidance on issues for which evidence is lacking

The initial consensus statement on the use of probiotics for the management of chronic/persistent diarrhea in children was not directly addressed because of the limited evidence. Rather than forego stating a recommendation, the expert panel recognized the need to include a recommendation based on clinical experience that results have been largely positive, with reductions in the duration and frequency of diarrhea. Areas where current evidence is lacking may be addressed in future updates as new information and new clinical studies become available.

### Cost and treatment-access considerations

New evidence has demonstrated the favorable efficacy and safety profile of *B. clausii*. However, the cost implications especially for developing Asian countries make it relatively inaccessible to many patients in the region.

Recommendations 5 (*Bacillus clausii* may be considered as adjunct to standard therapy in acute childhood diarrhea) and 9 (Probiotics administration may be considered for the prevention of AAD) help support treatment-access considerations in many resource-poor settings across the region.

## Conclusion

The Asian expert panel of pediatricians, pediatric gastroenterologists and a pediatric infectious disease specialist has developed a set of general recommendations on the use of probiotics as adjunct treatment in diarrhea, and the use of *Bacillus clausii* (O/C, SIN, N/R, T) in acute diarrhea, chronic diarrhea, and AAD to be used as reference in the management of pediatric diarrhea to improve patient outcomes. These recommendations consolidate the current evidence on the efficacy of probiotics in reducing the duration and severity of acute diarrhea in children, and the efficacy and safety of *B. clausii* (O/C, SIN, N/R, T) in reducing the duration and frequency of acute diarrhea.

## Data Availability

Not applicable.

## Appendix

**Table 8:** Summary of consensus statement voting and decisions using the 5-point Likert scale

Consensus statements and consensus voting	Decision
**1. Acute viral diarrhea is the best-established indication for probiotics administration in childhood. Probiotics have a promising role in the treatment of acute viral diarrhea.**	Accepted
Strongly Agree: 71%	
Agree: 29%	
Neither Agree or Disagree: 0%	
Disagree: 0%	
Strongly Disagree: 0%	
*Total (Strongly Agree and Agree): 100%*	
**2. Evidence has accumulated on the efficacy of probiotics in reducing the duration and severity of acute diarrhea in children.**	Accepted
Strongly Agree: 29%	
Agree: 57%	
Neither Agree or Disagree: 14%	
Disagree: 0%	
Strongly Disagree: 0%	
*Total (Strongly Agree and Agree): 86%*	
**3. Probiotics administration** ***should be considered*** **as an adjunct therapy for the prevention of antibiotic-associated diarrhea.**	Rejected: The original statement was revised to “Probiotics administration *may be considered* as an adjunct therapy for the prevention of antibiotic-associated diarrhea.” and another round of voting was done.
Strongly Agree: 14%	
Agree: 50%	
Neither Agree or Disagree: 7%	
Disagree: 21%	
Strongly Disagree: 7%	
*Total (Strongly Agree and Agree): 64%*	
**Probiotics administration may be considered as an adjunct therapy for the prevention of antibiotic-associated diarrhea.**	Accepted
Strongly Agree: 55%	
Agree: 45%	
Neither Agree or Disagree: 0%	
Disagree: 0%	
Strongly Disagree: 0%	
*Total (Strongly Agree and Agree): 100%*	
**4. Probiotics significantly reduce the risk of** ***Clostridium difficile*****-associated diarrhea in adults and children.**	Accepted
Strongly Agree: 31%	
Agree: 54%	
Neither Agree or Disagree: 0%	
Disagree: 15%	
Strongly Disagree: 0%	
*Total (Strongly Agree and Agree): 85%*	
**5.** ***Bacillus clausii*** **(O/C, N/R, SIN, TETRA) may be considered as adjunct to ORS and zinc in acute childhood diarrhea.**	Accepted
Strongly Agree: 54%	
Agree: 31%	
Neither Agree or Disagree: 15%	
Disagree: 0%	
Strongly Disagree: 0%	
*Total (Strongly Agree and Agree): 85%*	
**6.** ***Bacillus clausii*** **has been found to be safe in clinical trials conducted in Asian children with acute diarrhea.**	Accepted
Strongly Agree: 67%	
Agree: 33%	
Neither Agree or Disagree: 0%	
Disagree: 0%	
Strongly Disagree: 0%	
*Total (Strongly Agree and Agree): 100%*	
**7. Strain-specific poly-antibiotic–resistant** ***Bacillus clausii*** **is efficacious in reducing the duration and frequency of diarrhea, hospital stay, and financial burden.**	Accepted
Strongly Agree: 43%	
Agree: 43%	
Neither Agree or Disagree: 14%	
Disagree: 0%	
Strongly Disagree: 0%	
*Total (Strongly Agree and Agree): 86%*	
**8. There is very limited evidence supporting the use of probiotics for the management of chronic/persistent diarrhea in children.**	Accepted
Strongly Agree: 64%	
Agree: 36%	
Neither Agree or Disagree: 0%	
Disagree: 0%	
Strongly Disagree: 0%	
*Total (Strongly Agree and Agree): 100%*	
**9. Probiotics administration may be considered for the prevention of antibiotic-associated diarrhea (AAD).**	Accepted
Strongly Agree: 71%	
Agree: 29%	
Neither Agree or Disagree: 0%	
Disagree: 0%	
Strongly Disagree: 0%	
*Total (Strongly Agree and Agree): 100%*	
**10. Physicians should evaluate the risk factors for the occurrence of AAD or** ***Clostridium difficile*****-associated diarrhea, such as the class of antibiotics, duration of antibiotic treatment, need for hospitalization, age, comorbidities, and previous episodes of AAD or** ***C. difficile*****-associated diarrhea when considering probiotics for prevention of AAD in children.**	Accepted
Strongly Agree: 33%	
Agree: 53%	
Neither Agree or Disagree: 13%	
Disagree: 0%	
Strongly Disagree: 0%	
*Total (Strongly Agree and Agree): 86%*	
**11.** ***Bacillus clausii*** **can be used as co-adjuvant therapy for** ***Helicobacter pylori*** **eradication.**	Accepted
Strongly Agree: 20%	
Agree: 67%	
Neither Agree or Disagree: 13%	
Disagree: 0%	
Strongly Disagree: 0%	
*Total (Strongly Agree and Agree): 87%*	

**Table 9:** Summary of the characteristics and results of six randomized, controlled trials included in the review

Authors, Publication Year (Country)	Study Design	Intervention vs Comparator (Dosage and Duration)	Outcome Measures	Main Results
Canani et al., 2007 (Italy)	Prospective, multicenter, single-blind, randomized, controlled	1 × 10^9^ CFU of *Bacillus clausii* bid for 5 days + ORS for 3–6 h vs ORS for 3 to 6 h (followed by full strength formula of lactose or cows’ milk, depending on age, in both groups)	Total duration of diarrhea, number of stools/day and their consistency, incidence and median duration of vomiting, fever (> 37.5 °C), number of hospital admissions, safety and tolerability	Median duration of diarrhea in patients receiving *Bacillus clausii* (118 h) similar to control group (115 h), with an estimated difference of 1 h between both groups (*p* = 0.76). All other outcomes were also similar in both groups. *B. clausii* was well tolerated, with no observed adverse events.
Lahiri, 2008 (India)	Phase III, controlled, open-label, randomized, parallel-group, multicenter, comparative	2 × 10^9^ CFU of *B. clausii* bid + ORS + 20 mg/day of zinc supplement, for 5 days vs ORS + 20 mg/day of zinc supplement, for 5 days	Duration of diarrhea, mean number of daily stools, effect on consistency of stools, vomiting episodes per day, reported adverse events, parents’ overall global assessment of tolerability at end of treatment period	Mean (SD) duration of diarrhea lower in the experimental group (48.6 [38.2] h) vs control group (56.1 [40] h; *p* = 0.13). Difference in the mean (SD) number of stools until recovery statistically not significant (*p* = 0.19); trend favoring the experimental group (7.4 [6.5] motions vs 8.6 [6.5] motions in control group).
Lahiri, Jadhav et al., 2015 (India)	Open-label, prospective, randomized, controlled	2 × 10^9^ CFU of *B. clausii* bid + ORS + zinc, for 5 days vs ORS + zinc for 5 days	Mean duration of diarrhea, mean duration of hospitalization, frequency of diarrhea, direct and indirect costs	Mean duration of diarrhea 22.64 h and mean duration of hospital stay 2.78 days in the *B. clausii* group vs 47.05 h and 4.30 days, respectively, in the control group (*p* < 0.01 for diarrhea duration). Treatment with *B. clausii* reduced total treatment costs by 472 Indian rupees compared to ORS alone.
Lahiri, D’Souza et al., 2015 (India)	Open-label, prospective, randomized, controlled	2 × 10^9^ CFU of *B. clausii* bid + ORS + zinc, for 5 days vs ORS + zinc for 5 days	Mean duration of diarrhea, mean stool frequency, % of children with no dehydration, % of children benefiting from breastfeeding	Mean (SD) duration of diarrhea 22.26 h and mean stool frequency 1.15 in the *B. clausii* group vs 34.16 h and 1.70, respectively in control group (*p* < 0.05).
Maugo, 2012 (Kenya)	Randomized, double-blind, placebo- controlled	2 × 10^9^ CFU of *B. clausii* bid + ORS + zinc sulfate, for 5 days vs zinc sulfate + ORS + 1 vial bid of a placebo packaged in identical looking vials containing sterile water, for 5 days	Mean duration of diarrhea, mean duration of hospitalization, mean reduction of the number of diarrheal episodes per day	Mean (SD) duration of diarrhea in *B. clausii* group was shorter (77.59 [34.10] h) than placebo group (86.74 [40.16] h), with mean absolute difference between groups of 9.15 h (*p* = 0.248). Significant decrease in mean number of diarrheal motions on day 3 (2.74 [1.81] motions) in the *B. clausii* group vs 3.80 [2.70] motions in placebo group, mean absolute difference = 1.05 motions; *p* = 0.033) and day 4 (1.45 [1.13] motions in the *B. clausii* group vs 2.35 [2.19] motions in placebo group, mean absolute difference = 0.9 motions; *p* = 0.018) in the *B. clausii* group vs placebo group.
Urtula, Dacula, 2008 (The Philippines)	Monocentric, randomized, controlled	2 × 10^9^ or 4 × 10^9^ CFU of *B. clausii* per day, depending on the age of the children + ORS, for 3 days vs ORS for 3 days	Mean duration of diarrhea, mean duration of hospitalization, mean frequency of stools	Mean (SD) duration of diarrhea significantly shorter in the *B. clausii* group (69.84 [16.84] h) than in control group (83.76 [22.05) h] (*p* = 0.005), with absolute difference of duration of diarrhea between groups of 13.92 h. Mean duration of hospital stay was also shorter favoring *B. clausii* group (59.0 h vs 76.8 h) (*p* = 0.063).

*bid* twice-daily, *CFU* Colony-forming units, *ORS* Oral rehydration salts, *SD* Standard deviation

Adapted from: Ianiro G, et al. Nutrients. 2018;10:1074

## Abbreviations

AAD: Antibiotic-associated diarrhea
AGE: Acute gastroenteritis
BID: Bis in die (twice a day)
CDAD: *Clostridium difficile*-associated diarrhea
CDI: *Clostridium difficile* infection
CFU: Colony-forming unit
CI: Confidence interval
ESPGHAN: European Society for Pediatric Gastroenterology, Hepatology, and Nutrition
ESPID: European Society for Pediatric Infectious Diseases
GI: Gastrointestinal
ITT: Intention-to-treat
LGG: *Lactobacillus rhamnosus* GG
N/R: Resistant to novobiocin and rifampin
NNT: Number needed to treat
O/C: Resistant to chloramphenicol
ORS: Oral rehydration salts
RCT: Randomized, controlled trials
RR: Relative risk
SIN: Resistant to neomycin and streptomycin
T: Resistant to tetracycline
TID: Ter in die (three times a day)
WGO: World Gastroenterology Organisation
WHO: World Health Organization
